# MHC genes and parasitism in *Carassius gibelio*, a diploid-triploid fish species with dual reproduction strategies

**DOI:** 10.1186/1471-2148-13-122

**Published:** 2013-06-14

**Authors:** Andrea Šimková, Martin Košař, Lukáš Vetešník, Martina Vyskočilová

**Affiliations:** 1 Department of Botany and Zoology, Faculty of Science, Masaryk University, Kotlářská 2, Brno 611 37, Czech Republic; 2 Institute of Vertebrate Biology, Academy of Sciences of the Czech Republic, v.v.i., Květná 8, Brno 603 65, Czech Republic

**Keywords:** Gynogenesis, Sexual reproduction, Polyploid fish, MHC, Coexistence, Parasitism

## Abstract

**Background:**

The gibel carp is a fish species with dual reproduction modes, gynogenesis and sexual reproduction, coexisting in mixed diploid-polyploid populations. Following the Red Queen (RQ) assumption, asexual organisms are, due to their low genetic diversity, targets for parasite adaptation. Because MHC polymorphism is maintained by selection from parasites and sexual selection, MHC genes are considered as a suitable candidate for testing the RQ hypothesis. In this study, we investigated MHC variability and the selection pressure acting on MHC genes in sexual diploids and asexual triploids. In addition, we tested whether the asexual form of gibel carp suffers from higher parasite loads than the sexual form.

**Results:**

At the population level, genotype and allelic diversity of MHC were reduced in gynogenetic triploids when compared to sexual diploids. Different patterns in positively selected sites (PSS) between gynogens and sexual gibel carp were also found. A weak difference in parasite species richness was found between sexual fish and gynogens. However, two common clones of gynogens were significantly more parasitized than sexual diploids or other gynogens with rare MHC genotypes. At the individual level, the higher number of alleles was not associated with higher parasitism in either sexual diploids or gynogens.

**Conclusions:**

The differences in MHC diversity between gynogenetic triploids and sexual diploids are in accordance with the hypothesis of sexually-mediated selection increasing MHC diversity and fulfil a prerequisite of the Red Queen hypothesis. The different patterns in PSS between gynogens and sexual gibel carp also suggest the potential role of sexual selection and supports parasite-mediated selection maintaining MHC diversity. We showed that the most common MHC genotypes of gynogenetic triploids are the target of parasite selection. Our results suggest that the MHC genotype in gibel carp is more important than allelic number for immunocompetence.

## Background

The coexistence of asexual and sexual forms in nature has been reported mainly in plants and invertebrates. Concerning vertebrates, asexual reproduction in many forms has been found in fishes, amphibians and lizards. The mechanisms promoting the coexistence of asexual fish living in sympatry with sexual ones have been investigated e.g. *Phoxinus*[1], *Poecilia*[2], *Carasssius auratus*[3].

Sexual and asexual forms do not coexist easily. Sexual forms suffer from the twofold costs of sex (i.e. the twofold disadvantage of producing males or the two fold cost of meiosis) and, therefore, they should be outcompeted by asexual forms using the same resources and producing eggs at twice the rate [4,5]. On the other hand, the long-term costs associated with the accumulation of deleterious mutation leading to fitness decline in asexual forms and their potential extinction within many generations have been predicted [6]. Moreover, asexual forms have a limited ability to adapt to environmental changes [2], show reduced performance under stress [7], and exhibit a higher susceptibility to parasites when compared to sexual forms (reviewed by Hamilton *et al*. [8] and shown by empirical studies (e.g. [1,9])). Concerning the form of asexual reproduction termed gynogenesis, the offspring are formed parthenogenetically but egg development is induced by sperm of the same or closely-related sexual species. Gynogenetic females can only persist in sympatry with closely-related bisexual forms. Thus, this form of reproduction has some of the disadvantages of sexual and asexual reproduction [10,11].

One of the most widely accepted hypotheses proposed to explain the maintenance and evolution of sex is the Red Queen hypothesis [5,12-14]. This hypothesis postulates antagonistic coevolution between hosts and parasites leading to sustained oscillations in genotype frequencies, whereby hosts refine their defense strategies against parasites, and parasites, in turn, evolve counter-adaptations to these defense strategies. The core idea is that parasites are generally under selection to infect the most common genotypes in the local host population. Parasite mediated frequency-dependent selection leads to genetic polymorphism in both the host and parasite [15]. In species where asexual reproduction is possible (i.e. the asexual clone becomes the most common genotype in the host population), co-evolutionary interactions with parasites may select for sexual reproduction in hosts as a way of reducing the risk of infection in offspring [8,14]. Thus, the high level of parasite infection in common asexual clones could favor genetically diverse sexual individuals and promote the short-term coexistence of sexual and asexual populations. In fish, several studies were conducted to compare the level of parasitism between sympatric asexual and sexual forms or species. Some of them support the Red Queen hypothesis and showed that asexual fish suffer from a higher parasite load than sexual fish (e.g. [1,3,16]). However, no difference in parasitism between asexual and sexual forms of fish species was detected in several empirical studies (e.g. [9,17]).

The MHC is a family of highly polymorphic genes occurring in vertebrates that encode cell-surface glycoproteins responsible for the recognition of peptide fragments of self or non-self-origin and for presenting them to T cells, which can initiate an appropriate immune response resulting in the destruction of antigen-presenting cells or in antibody production [18]. The high polymorphism is most pronounced in the peptide-binding region (PBR), which is at specific amino-acid sites directly in contact with the bound peptides. MHC polymorphism is maintained by balancing selection driven by parasites or mating choice [19-21]. For this reason, MHC is a suitable candidate for testing the mechanisms proposed for the coexistence of asexual and sexual species based especially on the Red Queen hypothesis. The difference in the specific immunity, of which MHC is representative, may cause negative density-dependence mortality in the asexual form and thus maintain sexual reproduction in the populations. The variability of MHC in asexual vertebrates has only been examined by a few studies [10,22]. To our knowledge, parasite-mediated selection acting on MHC diversity has not yet been compared between sexual and asexual fish.

Gibel carp (*Carassius gibelio*) represents an interesting and unique species in which the coexistence of gynogenetic reproduction and gonochoristic reproduction is known. The origin of this species is thought to be eastern Russia and it colonized Europe successively during the 20th century [23]. In 1975, a non-native gibel carp permeated into the Czech hydrologic system by migration from the Danube River and, with human help and by its own invasive activities, gradually colonized most of the suitable biotopes of the three Czech basins. The former populations of *C. gibelio* were composed of triploid females with gynogenetic reproduction in which gynogenetic females used the sperm of males of other cyprinid fish species (i.e. *Carassius carassius*, *Cyprinus carpio* and *Abramis brama*) for the activation of egg development [24,25]. The solely female character of populations had been recorded up to 1992, when the first males were recorded in the populations. A few years later, *C. gibelio* had formed stable mixed diploid-polyploid populations composed of sexually reproducing diploids, asexually reproducing dominant triploid females, and very rare triploid and tetraploid males [26]. Following the different advantages and disadvantages related to gynogenesis and sexual reproduction in gibel carp, a few studies [27-29] were performed to compare growth as an important life trait between diploids and triploids expecting that gynogenetic triploids should exhibit fast growth than sexually reproducing diploids. However, no difference in growth patterns between two forms was found. The analyses of the control region and cytochrome *b* gene showed that both sexually reproducing diploids and gynogenetic triploid females of morphologically identified *C. gibelio* in the Czech Republic have the same origin [30]. Gibel carp is parasitized by a wide range of metazoan parasites [31]), many of them (especially Monogenea) are specific to *C. gibelio* and the phylogenetically closely related species *C. carassius*. For host specific parasites, long-term host-parasite coevolution (resulting in parallel changes to the host immune system) is hypothesized and such parasites are primarily responsible for the evolution of MHC polymorphism [32]. In addition, gill and skin monogeneans represent the species richest group; they are pathogenic to gibel carp and closely-related fish species, causing fish mortality especially in fish aquaculture (e.g. [33,34]).

This study was focused on the variability of MHC genes and parasitism in two forms of gibel carp with different modes of reproduction and ploidy level. First, we compared MHC variability and the selection pressure acting on MHC genes in sexual diploids and asexual triploids. We expected that differences between sexual and asexual forms would be due to the negative frequency dependent selection acting in asexually reproducing females (i.e. following the Red Queen hypothesis, low genotypic diversity makes asexual organisms an ideal target for parasite adaptation) and also due to the sexual selection acting on MHC diversity in sexual diploids. We addressed the following specific questions 1) Do sexual gibel carp exhibit a higher diversity of MHC genotypes than asexual gibel carp? 2) Do asexual gibel carp (or at least common asexual MHC genotypes) suffer from higher parasite loads than sexual gibel carp?

## Results

The mixed population of gibel carp in the locality studied was composed of 60% triploid individuals and 40% diploid individuals (with a male–female ratio of 1:1) with no variation between two consecutive years. Approximately the same number of triploid and diploid individuals (Table 1), all of them with a similar total body size, were analyzed (19.47 ± 2.94 cm).

**Table 1:** **Comparison of the number of *DAB *alleles and diversity (mean and SE for overall nucleotide distance using Jukes-Cantor and overall amino-acid distance using Poisson correction are shown) in diploid and triploid forms of gibel carp**

	**Sexual diploids**	**Gynogenetic triploid females**
Number of analyzed individuals	44	47
Number of total *DAB*-like sequences	22	15
Number of *DAB1*-like sequences	10	6
Number of *DAB3*-like sequences	12	9
Overall nucleotide diversity	0.260 (0.024)	0.264 (0.024)
Overall amino-acid diversity	0.489 (0.060)	0.475 (0.057)
Nucleotide diversity using *DAB1*-like sequences	0.103 (0.011)	0.090 (0.012)
Nucleotide diversity using *DAB3*-like sequences	0.118 (0.014)	0.112 (0.014)
Amino-acid diversity using *DAB1*-like sequences	0.222 (0.033)	0.204 (0.034)
Amino-acid diversity using *DAB3*-like sequences	0.239 (0.038)	0.231 (0.036)

### Variability of *DAB* genes (MHC IIB class)

Different genotype diversity was found for gynogenetic triploids and sexual diploids. Diploids expressed 32 genotypes whilst triploids expressed only 8 genotypes (Table 2). Two genotypes were common in triploids, i.e. 53% of triploid females expressed the genotype including Cagi-DAB3*05 and Cagi-DAB3*34 alleles (termed as common genotype “A”) and 26% of triploid females expressed the genotype including Cagi-DAB1*27, Cagi-DAB3*16 and Cagi-DAB3*26 alleles (termed as common genotype “B”). Other triploids expressed rare genotypes. All genotypes of diploids were rare, i.e. expressed by only one or two individuals except for the genotype including one allele Cagi-DAB1*17 found in 7 diploids.

**Table 2:** **The frequency (i.e. N as a number of individuals expressing a given genotype) and allelic profile (i.e. the presence of each *DAB *allele) of each genotype (3n or 2n) are shown**

	***Cagi-DAB1***												***Cagi-DAB3***																	
**N**	*03	*05	*09	*11	*17	*21	*22	*23	*24	*27	*29	*33	*05	*07	*09	*11	*12	*13	*14	*15	*16	*19	*20	*21	*23	*24	*26	*29	*33	*34
3n																														
25													x																	x
12										x											x						x			
3																							x					x		
2		x	x															x												
2	x			x																										
1		x	x																									x		
1					x																									
1																x										x				
2n																														
7					x																									
3					x	x																								
3															x															
1		x	x														x													
1					x																				x					
1					x				x											x										
1						x																								
1						x	x																							
1							x		x																					
1									x											x									x	
1									x							x				x										
2											x																			
1					x						x																			
1							x		x		x																			
1											x											x								
1											x														x					
1											x				x															
1																			x											
1																													x	
2															x							x								
1					x		x																							
1						x																		x						
1							x																							
1								x								x														
1									x											x										
1					x					x	x																			
1										x	x			x																
1												x			x															
1															x				x											
1																									x					
1													x																	x
1														x	x									x						

The individuals of both sexual diploids and gynogenetic triploids expressed genotypes including the alleles of a single lineage (*DAB1*-like or *DAB3*-like) or the alleles of both lineages *DAB1*-like and *DAB3*-like. Due to the expression of the common genotype “A” of the *DAB3*-like lineage in gynogenetic triploids, 62% of triploid individuals expressed the genotypes belonging to a single *DAB3*-like lineage. Meanwhile, 32% of triploid individuals expressed the combined genotypes, whilst only 6% expressed the genotypes of the *DAB1*-like lineage. A different ratio of genotypes was found in diploids, where 45% of diploid individuals expressed the genotypes of the *DAB1*-like lineage, 25% of diploid individuals expressed *DAB3*-like genotypes, and 30% expressed the combined genotypes (*DAB1*-like and *DAB3*-like).

At population level, the total number of alleles expressed by sexual diploids and gynogenetic triploids was different (Tables 1 and 2). A total of 30 different alleles were recorded in the population of gibel carp investigated, but only 7 alleles were shared between the two forms (Table 2). The majority of alleles were specific to either diploid or triploid genotypes. Three alleles shared by sexual diploids and gynogenetic triploids were expressed in common genotypes of triploids, and one shared allele was the most frequent allele found in diploid genotypes. Even if the diploids expressed a wide range of different genotypes, three alleles were more frequent than others i.e. Cagi-DAB1*17, Cagi-DAB1*29 and Cagi-DAB3*09 (Table 2). No significant influence of year of collection on the number of alleles expressed by individuals was found (MW test, p > 0.05). Therefore, data from two years were pooled for the next analyses. The number of alleles per individual (both diploids and triploids) ranged from 1 to 3 *DAB* alleles (Table 2). The number of alleles per individual expressed by triploids was significantly higher than the number of alleles expressed by diploids (MW test, p = 0.002).

Even the total number of different *DAB* sequences was higher for sexual diploids than for gynogenetic triploids (approximately 1.6 times) at the population level; the mean overall nucleotide diversity was similar for both forms. After translating the nucleotide sequences into amino-acid sequences, the alignment reflected the high level of non-synonymous substitutions in *DAB1*-like and *DAB3*-like genes in both forms. The rate of nonsynonymous substitutions was significantly higher compared to synonymous ones (Table 3).

**Table 3:** **The estimated rates (mean and standard error) of non-synonymous (*d***_**N**_**) and synonymous substitutions (*d***_**S**_**), and the Z-test of positive selection (test statistics *d***_**N**_**-*d***_**S**_**) with *p*-value**

**Form**	***DAB *gene**	***d***_**S**_	***d***_**N**_	***d***_**N **_***- d***_**S**_	***p***
triploid	*DAB1-like* (6)	0.043 (0.015)	0.104 (0.018)	2.742	0.004
	*DAB3-like* (9)	0.057 (0.019)	0.129 (0.023)	2.769	0.003
diploid	*DAB1-like* (10)	0.057 (0.016)	0.116 (0.018)	2.743	0.004
	*DAB3-like* (12)	0.062 (0.020)	0.132 (0.024)	2.845	0.003

Standard errors estimates shown in parentheses were obtained from 1000 bootstrap replicates. Numbers following *DAB* gene groups shown in parentheses indicate the number of different allelic variants in the alignment.

Mean amino-acid variability calculated for the total data set at the population level was slightly higher for sexual diploids than for gynogenetic triploids (Table 1). A similar nucleotide and amino-acid diversity for the *DAB1*-like gene and for the *DAB3*-like gene was found when comparing both forms. However, the means of nucleotide and amino-acid diversity were higher in the *DAB3*-like data set than in the *DAB1*-like data set (Table 1). At the individual level, gynogenetic triploids had a significantly higher amino-acid diversity than sexual diploids (t-value = 2.404, df = 89, p = 0.018). However, when corrected for the number of alleles (using residual analysis) no significant difference in amino-acid diversity was found between gynogenetic triploids and sexual diploids (t-test, p > 0.05). Nucleotide diversity was not significantly different between the two forms of gibel carp (t-test, p = 0.089). However, the variance in both amino-acid diversity and nucleotide diversity was significantly higher in sexual diploids compared to gynogenetic triploids without correction for the number of alleles (p < 0.001) and also using correction for the number of alleles (p < 0.001).

### Positive selection in *DAB* sequences

**Figure 1:**
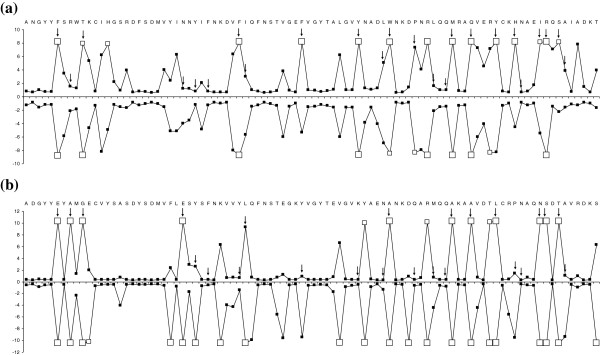
**Approximate posterior means of ω, calculated as the weighted average of ω over the 11 site classes, weighted by the posterior probabilities under the random sites model M8 (β and ω) are shown for *DAB1*-like sequence variants (a) and for *DAB3*-like sequence variants (b).** The values for sexual diploids are shown above the *x*-axis and those for gynogenetic diploids are shown below the *x*-axis. Sites inferred to be under positive selection at the 99% level are indicated by large white squares and those at the 95% level are indicated by medium white squares. Arrows (showed only above x-axis) indicate the positions of human antigen binding sites (HLA-*DRB1* gene) [35]. The reference sequences used in the graphic presentation are the following: Cagi-DAB1*05 for *DAB1*-like sequence variants and Cagi-DAB3*05 for *DAB3*-like sequence variants.

Maximum likelihood estimates of parameters under different codon models of variable ω across sites and the number of PSS for both forms of gibel carp are included in Table 4. The LRT statistic comparing the two models indicates that the alternative models M2a, M3 and M8 (i.e. the models that account for the sites under positive selection) fit the data significantly better (p < 0.001) than the simpler models M1a, M0 and M7 (i.e. the models that do not allow for positive selection), which indicates the action of positive selection at specific sites in *DAB* sequences. Bayes identification of sites under positive selection calculated using the M8 model is included in Figure 1.

**Table 4:** **Log-likelihood values and parameter estimates under random-site models for two groups *DAB1 *and *DAB3 *in gynogenetic triploids and sexually reproducing diploids of *Carassius gibelio***

**Fish - *DAB*-like gene**	**Model code**	**Log-likelihood**	**Estimates of parameters**	**Number of PSS**
Triploids - *DAB1-*like	M0: one ratio (1)	−1707.7	ω = 2.17	
	M3: discrete (5)	−1570.8	p0 = 0.48, **p1 = 0.43**, (**p2 = 0.09**),	not analyzed
			ω0 = 0.31, **ω1 = 4.80**, **ω2 = 35.44**	
	M1a: nearly neutral (1)	−1660.59	p0 = 0.48, (p1 = 0.52), (ω0 = 0.07), (ω1 = 1)	not allowed
	M2a: positive selection (3)	−1587.79	p0 = 0.30, p1 = 0.57, (**p2 = 0.13**)	8*, 3**
			ω0 = 0, (ω1 = 1), **ω2 = 10.50**	
	M7: beta (2)	−1662.59	p = 0.13, q = 0.1	not allowed
	M8: beta and ω (4)	−1558.13	p0 = 0.87, (**p1 = 0.13**)	11*, 8**
			p = 0.03, q = 0.02, **ω = 10.22**	
Triploids - *DAB3*-like	M0: one ratio (1)	−1088.36	ω = 2.99	
	M3: discrete (5)	−992.13	p0 = 0.67, **p1 = 0.26**, (**p2 = 0.07**),	not analyzed
			ω0 = 0.45, **ω1 = 12.72**, **ω2 = 66.25**	
	M1a: nearly neutral (1)	−1056.48	p0 = 0.59, (p1 = 0.41), (ω0 = 0.02), (ω1 = 1)	not allowed
	M2a: positive selection (3)	−1002.56	p0 = 0.30, p1 = 0.44, (**p2 = 0.26**)	21*, 16**
			ω0 = 0, (ω1 = 1), **ω2 = 17.36**	
	M7: beta (2)	−1056.89	p = 0.01, q = 0.02	not allowed
	M8: beta and ω (4)	−1002.69	p0 = 0.74, (**p1 = 0.26**)	22*, 21**
			p = 0.01, q = 0.005, **ω = 19.60**	
Diploids - *DAB1-*like	M0: one ratio (1)	−995.79	ω = 2.30	
	M3: discrete (5)	−968.41	p0 = 0.14, p1 = 0.57, (**p2 = 0.29**),	not analyzed
			ω0 = 0, ω1 = 1, **ω2 = 8.71**	
	M1a: nearly neutral (1)	−989.53	p0 = 0.39, (p1 = 0.61), (ω0 = 0.03), (ω1 = 1)	not allowed
	M2a: positive selection (3)	−968.41	p0 = 0.14, p1 = 0.57, (**p2 = 0.29**)	13*, 8**
			ω0 = 0, (ω = 1), **ω2 = 8.71**	
	M7: beta (2)	−989.58	p = 0.03, q = 0.02	not allowed
	M8: beta and ω (4)	−968.41	p0 = 0.71, (**p1 = 0.29**)	15*, 11**
			p = 0.02, q = 0.01, **ω = 8.66**	
Diploids - *DAB3-*like	M0: one ratio (1)	−1343.98	ω = 2.96	
	M3: discrete (5)	−1193.40	p0 = 0.65, **p1 = 0.27**, (**p2 = 0.08**),	not analyzed
			ω0 = 0.31, **ω1 = 8.47**, **ω2 = 56.35**	
	M1a: nearly neutral (1)	−1285.98	p0 = 0.60, (p1 = 0.39), (ω0 = 0.03), (ω1 = 1)	not allowed
	M2a: positive selection (3)	−1207.25	p0 = 0.43, p1 = 0.41, (**p2 = 0.16**)	13*, 11**
			ω0 = 0, (ω = 1), **ω2 = 13.52**	
	M7: beta (2)	−1286.2	p = 0.02, q = 0.02	not allowed
	M8: beta and ω (4)	−1207.26	p0 = 0.84, (**p1 = 0.16**)	14*, 11**
			p = 0.005, q = 0.005, **ω = 13.64**	

The number following the model code given in parentheses represents the number of free parameters for the ω ratios. Parameters indicating positive selection are presented in bold type. Parameters in parentheses are presented for clarity only but are not free parameters; for example, under M8 *p*1 = 1-*p*0; PSS are the positive selected sites identified using the BEB method at *: P > 95%; **: P > 99%; ω is the selection parameter; p_n_ is the proportion of sites that fall into the ω_n_ site classes; p and q are the shape parameters of the β function (for M7 and M8 models).

The estimates from the M8 model suggest a different pattern of selection in *DAB1*-like and *DAB3*-like genes between both forms, i.e. a different number and different positions of PSS in sexual diploids and gynogenetic triploids were found (Figure 1). Concerning sexual diploids, a similar number of PSS sites were under positive selection (significance thresholds of p < 0.05) in the *DAB1*-like and *DAB3*-like sequences (Table 4). 13 out of 15 PSS identified in *DAB1*-like sequences and 11 out of 14 PSS identified in *DAB3*-like sequences corresponded to human antigen binding sites (ABS) identified using crystallography by Brown *et al.*[35] (see Figure 1). For *DAB* sequence variants of gynogenetic triploids, 11 sites were identified as targets of positive selection in *DAB1*-like alignment (p < 0.05), 9 of them identical to human ABS. A higher number of PSS were detected in *DAB3*-like sequences of gynogenetic females compared to the PSS identified in *DAB1*-like sequences in this form or PSS identified in *DAB1*-like or *DAB3*-like sequences of sexual diploids. 13 out of 22 PSS identified in *DAB3*-like sequences of gynogenetic triploids corresponded to human ABS. All PSS found in *DAB3*-like sequences in sexual diploids were also identified as PSS in *DAB3*-like sequences of gynogenetic triploids. 9 PSS found in *DAB1*-like sequences of gynogenetic triploids were identified in *DAB1*-like sequences of diploids.

### MHC and parasitism

No significant difference in parasite species richness was found between the fish investigated in 2005 and 2006 (t-test, p > 0.05). Therefore, the parasitological data were pooled for all analyses. A total of 16 metazoan parasite species were found in the gibel carp studied. Among them, Monogenea was the species richest group (Table 5). The prevalences of five *Dactylogyrus* species were high (more than 80%) and the prevalences of another three ectoparasite species *D. vastator*, *G. sprostonae* and glochidum were moderate (20-33% considering all gibel carp). The presence of other parasite species was rare (a prevalence of less than 5% considering all fish individuals). Means of parasite species richness, total parasite abundance, abundance of *Dactylogyrus*, and parasite diversity are shown in Table 6. When comparing infection by parasite species between the two forms of gibel carp (Table 5), the prevalences of the five dominant *Dactylogyrus* species were similar. The intensities of infection for four of the five dominant *Dactylogyrus* species tended to be higher in triploids than in diploids. Moreover, triploids were occasionally parasitized by four rare endoparasite species that were absent in diploids (Table 5).

**Table 5:** **Prevalence and parasite abundance (median, minimum-maximum and percentile boundaries 5%-95%) for parasite species in *Carassius gibelio***

			**Diploids (N = 44)**				**Triploids (N = 47)**			
	**Parasite species**	**HS**	**Prevalence**	**Abundance**			**Prevalence**	**Abundance**		
				**Median**	**Min-max**	**Percentiles**		**Median**	**Min-max**	**Percentiles**
Monogenea	*Dactylogyrus anchoratus*	Sph	93.2%	14.74	0-55	0-42	91.5%	14.24	0-86	0-74
	*Dactylogyrus baueri*	Sc	-	-	-	-	2.1%	0	0-1	0
	*Dactylogyrus dulkeiti*	Sc	100%	35.12	4-137	8-83	100%	32.35	1-158	4-113
	*Dactylogyrus formosus*	Sc	95.5%	7.48	0-68	1-49	83%	9.55	0-72	0-59
	*Dactylogyrus inexpectatus*	Sc	95.5%	12.01	0-149	3-60	95.7%	18.64	0-77	2-54
	*Dactylogyrus intermedius*	Sc	100%	23.63	3-142	4-114	100%	32.23	8-183	10-108
	*Dactylogyrus vastator*	Sph	18.2%	0	0-7	0-5	46.8%	0	0-6	0-6
	*Gyrodactylus sprostonae*	Sph	27.3%	0	0-25	0-5	27.7%	0	0-5	0-3
	*Gyrodactylus longoacuminatus*	Sph	2.3%	0	0-2	0	6.4%	0	0-2	0-1
Mollusca	glochidia (larv. stages of *Unio* and *Anodonta*)	G	25%	0	0-21	0-6	38.3%	0	0-12	0-5
Nematoda	*Philometroides sanguinea*	Sc	2.3%	0	0-1	0	2.1%	0	0-1	0
	*Schulmanela petruschewskii*	G	-	-	-	-	4.3%	0	0-75	0
Digenea	Digenea sp.	not evaluated	-	-	-	-	8.5%	0	0-5	0-4
Cestoda	Caryophyllaceae sp.	G	-	-	-	-	2.1%	0	0-1	0
Acanthocephala	*Acanthocephalus anguillae*	G	4.5%	0	0-3	0	4.3%	0	0-10	0
Hirudinea	*Hemiclepsis marginata*	G	-	-	-	-	2.1%	0	0-1	0

Sc – specific parasite i.e. parasitizing two congeneric *C. gibelio* and *C. carassius*, Sph – specific parasite i.e. parasitizing phylogenetically related species *C. gibelio*, *C. carassius* and *Cyprinus carpio*. G – generalist (non-specific) parasite species. Host specificity (HS) was taken from Moravec [31].

**Table 6:** Parasite load in sexual diploids and gynogenetic triploids of gibel carp (mean and SD are shown)

	**Sexual diploids**	**Gynogenetic triploids**
Parasite species richness	5.57 ± 1.05	6.04 ± 1.35
Total parasite abundance	128.23 ± 88.72	156.13 ± 115.37
*Dactylogyrus* abundance	125.66 ± 88.03	152.13 ± 112.25
Brillouin index diversity	1.34 ± 0.17	1.36 ± 0.23

First, when fish MHC was expressed as the number of *DAB* alleles, nested ANOVA revealed no significant effects of fish MHC and reproductive strategy on parasite load measured by parasite species richness or parasite abundance (p > 0.05). Next, when fish MHC was expressed as the type of *DAB* lineage, nested ANOVA (total F = 2.319, p = 0.05) revealed a statistically significant effect exerted by fish MHC (F_2.85_ = 3.78, p = 0.027) and an insignificant effect exerted by reproduction strategy (F_3.85_ = 0.983, p = 0.404) on parasite load expressed as parasite species richness. Using the Bonferonni post-hoc test, a significant difference was found in parasite species richness only between the *DAB3* and *DAB1* genotypes (p = 0.029), where the *DAB3* genotype was more parasitized than the *DAB1* genotype. No statistical difference between the genotype of combined lineages (i.e. *DAB1* and *DAB3*) and single genotypes (*DAB1* or *DAB3*) was found, demonstrating no significant link between increasing MHC variability and parasite load.

**Figure 2:**
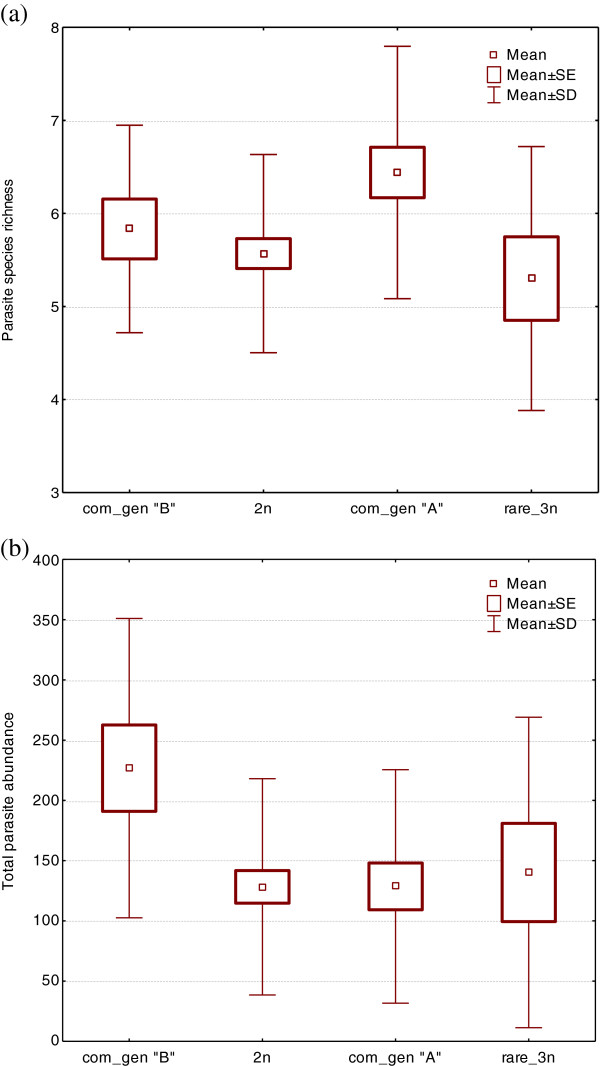
Parasite species richness (a) and total parasite abundance (b) in sexual diploids (2n), rare gynogenetic triploids (rare_3n), gynogenetic triploids with common genotype “A” (com_gen “A”) and gynogenetic triploids with common genotype “B” (com_gen “B”).

To test the Red Queen hypothesis, first, parasitism was compared between gynogenetic triploids and sexual diploids. A higher mean parasite species richness was found in gynogenetic triploids compared to sexual diploids (Table 6). However, the difference in parasite species richness between gynogenetic triploids and sexual diploids was not statistically significant (t-test, p = 0.069). No significant difference in parasite abundance or parasite diversity was found between diploids and triploids (t-test, p > 0.05). No significant difference was found when comparing the variance in parasite abundance or parasite diversity (F-test of variances, p > 0.05). Next, we divided the MHC genotypes of triploid individuals into three groups: common genotype “A” (N = 25), common genotype “B” (N = 12), and rare genotype (N = 10). The parasitism of each common genotype was compared to the parasitism of gynogenetic triploids with the rare genotype and sexual diploids (Figure 2). Significantly higher parasite species richness was found in gynogenetic triploids expressing the common genotype “A” than in sexual diploids (t-value = 2.955, df = 67, p = 0.004) and gynogenetic triploids with the rare genotype (t-value = 2.218, df = 33, p = 0.033). However, after Bonferroni correction, only the difference between triploids expressing common genotype “A” and sexual diploids was significant (p = 0.016). No difference in parasite abundance or parasite diversity was found between gynogenetic triploids expressing the common genotype “A” and sexual diploids or gynogenetic triploids with the rare genotype (t-test, p > 0.05). A significantly higher parasite abundance was found for triploid individuals with common genotype “B” when compared to sexual diploids (t-value = 2.419, df = 54, p = 0.019) or gynogenetic triploids with common genotype “A” (t-value = 2.150, df = 35, p = 0.039). After Bonferroni correction, no relationship was significant (p > 0.05). *Dactylogyrus* species represented 56% of total parasite species and 98% of total parasite abundance. A significantly higher *Dactylogyrus* abundance was found for triploids with common genotype “B” when compared to sexual diploids (t-value = 2.476, df = 54, p = 0.016) or gynogenetic triploids with common genotype “A” (t-value = 2.213, df = 35, p = 0.034). After Bonferroni correction, only the difference between gynogenetic triploids with common genotype “B” and sexual diploids was significant (p = 0.048).

## Discussion

This study presents the first analysis of the genetic diversity of functional MHC class IIB genes in gibel carp, a fish species with dual reproduction modes – sexual reproduction in diploids and gynogenesis in triploid females. Similarly to previous studies analyzing *DAB* genes in cyprinid fish (e.g. [36-38]), high MHC IIB diversity was also shown in this diploid-polyploid fish species. Both forms express the alleles of one locus (*DAB1*-like or *DAB3*-like) or the alleles of both loci (*DAB1*-like and *DAB3*-like), which was shown for diploid cyprinid species using genomic or expressed MHC IIB genes (e.g. [36,37,39]).

Our study revealed the presence of many alleles specific to gynogenetic triploids or specific to sexual diploids. Moreover, several alleles were found at high frequencies only in triploids (associated with the presence of two common genotypes) or only in diploids. Concerning the specific and frequent alleles found in gynogenetic triploids, we cannot distinguish if they are merely characteristics of our study population, or if they have persisted ever since the species invasion of the Czech Republic, or even since the origin of the triploid form (possibly through a hybridization event). Such hypotheses could be evaluated using a large sample and time series from a wide range of gibel carp populations.

Despite the reduced genotype diversity in triploids (see below), similar overall nucleotide and amino-acid diversity at the population level was found for *DAB* datasets in sexual diploids and gynogenetic triploids, which indicates the genetic variation among different gynogenetic lineages of gibel carp. At the individual level, gynogenetic triploids expressed mainly 2 or 3 *DAB* alleles (only a single triploid individual expressing 1 allele was identified in our sample), whilst sexual diploids expressed mainly 1 or 2 *DAB* alleles. This could be explained by polyploidization of the genome, where a higher number of gene copies is expected for polyploid individuals, which might give them a crucial advantage in pathogen recognition. However, following the Red Queen hypothesis, asexually reproducing organisms should be very susceptible to pathogen infection because of low genotypic diversity which could annihilate the positive effect of having many MHC copies [10]. When compared *Carassius gibelio* to *Poecilia*, between 1 and 4 MHC class IIB sequences per individual were found in both asexual Amazon molly (*P. formosa*) and sexual Sailfin molly (*P. latipinna*) [22] (biologically, these species are considered as two forms of the unisexual-bisexual species complex of mollies). Thus, it seems that the high number of MHC IIB variants in gynogenetic individuals does not represent a general rule for the species where two forms with different reproduction modes coexist. The number of MHC alleles per individual may be determined by the potential duplication of some MHC lineage found in cyprinid species e.g. duplication of the *DAB1* lineage in common carp *(Cyprinus carpio*) [39], duplication of the *DAB3* lineage in chub *(Squalius cephalus*) [36], or duplication of both *DAB1* and *DAB3* lineages found only for the diploid form of gibel carp in this study. It is also possible that some alleles or allelic lineages are not detectable in cDNA, as they are not expressed.

High MHC polymorphism is explained by two, though not mutually exclusive hypotheses, i.e. sexual selection and parasite-mediated selection. We tested whether or not the sexual form of gibel carp shows higher MHC polymorphism than the gynogenetic form, following the hypothesis that MHC diversity is maintained by sexual-mediated selection. Schaschl *et al*. [22] presumed that high MHC diversity in the asexual form or comparable diversity between sexual and asexual forms indicates that sexual selection is not important for explaining MHC diversity. Less MHC diversity at the allelic as well as genotypic level for asexual species compared to sexual ones was also hypothesized by Lampert *et al*. [10] as an important prerequisite of the Red Queen hypothesis.

In our study, the high MHC genotype and allelic diversity (i.e. number of genotypes and number of allelic copies at the population level) in sexual diploids compared to gynogenetic triploids living in the same habitat is consistent with the prediction that MHC diversity is driven by sexually-mediated selection (i.e. meiotic recombination generates and maintains new MHC alleles in sexual diploids). On the other hand, reduced MHC genotype diversity in gynogenetic triploids is probably due to the lack of recombination and segregation in these asexually reproducing organisms, where only point mutations could generate new MHC alleles. Similar findings were previously shown in *Poecilia*, i.e. lower MHC genotype diversity and the reduced diversity of MHC class I and class IIB alleles was found in asexual Amazon molly compared to sexual Sailfin molly [10,22]. Lower diversity in MHC class I genes was also found in a parthenogenetic gecko species compared to closely related sexual gecko species [40]. The difference in genotype and allelic diversities between asexually and sexually reproducing gibel carp could be the result of sexual selection and/or selection from pathogens and parasites, or may be due to genetic drift or the bottleneck effect after introduction, as was suggested for asexual Amazon mollies by Schaschl *et al*. [22].

Our study revealed a similar number of positively selected sites in both *DAB1*-like and *DAB3*-like genes in sexual diploids, where the majority of PSS were identical with human antigen binding sites. However, when analyzing the selection pattern in *DAB* genes of gynogenetic triploids, the different number of PSS between *DAB1*-like and *DAB3*-like sequence alignments was shown. This may suggest that sexual selection or parasite-mediated selection (or, indeed, both of them) contribute to the maintenance of MHC diversity in diploid-polyploid populations of gibel carp. However, we should emphasize that only extensive experimental study could clarify the mechanisms of selection affecting MHC diversity in gibel carp. In addition, we showed stronger positive selection in *DAB3*-like genes in gynogenetic triploids compared to the selection in *DAB1*-like genes in gynogenetic triploids or to the selection in sexual diploids, which suggests a potential functional difference between *DAB1*-like and *DAB3*-like genes (likely linked to immune defense), especially for triploids.

Parasite-mediated selection (i.e. negative frequency dependent selection) together with the coevolutionary “arms races” between hosts and parasites form the core idea of the Red Queen hypothesis. We showed that, at the population level, MHC genotype and allelic diversities in asexual triploids are reduced compared to sexual diploids. Therefore, gibel carp clearly fulfills a prerequisite of the Red Queen hypothesis underlying the evolution of sex. Concerning parasites, their virulence and pathogenicity represent important components of the Red Queen hypothesis. These features were demonstrated for some gill and skin monogeneans (e.g. [41,42]). *Dactylogyrus* parasitizing *Carassius* species and closely related common carp are considered to be the most pathogenic monogenean species on the gills causing fish mortality [33,34]. We evaluated parasite load, including metazoan parasite species with a high prevalence of *Dactylogyrus* species that are host specific to *Carassius* species or specific to phylogenetically closely related fish species (i.e. *Carassius* species and *Cyprinus carpio*).

In our study, the parasite load on gynogenetic and sexual forms was compared following the prediction of the Red Queen hypothesis, i.e. sex provides an advantage to hosts by creating rare genotypes that confer resistance to parasites. Thus, in the case when the genetic diversity of asexual lineages is low, the asexually reproducing form is more intensely infected than the sexual form [3,16]. Our study showed that parasite species richness is slightly higher in the gynogenetic form compared to the sexual form. The finding that the overall amino-acid diversity of *DAB* genes in gynogens was comparable to the amino-acid diversity in the sexual form suggests that the analyzed gynogenetic lineages are genetically diverse. Therefore, the subsequent analyses were focused on the parasite load of common MHC IIB genotypes in gynogenetic triploids, following the prediction that the parasite load of the sexual form should be lower than that of the more common clone, but that the parasite load of some infrequent clones might not necessarily be higher than that of the sexual form [16]. Two common MHC IIB genotypes were more parasitized than the genotypes of sexual diploids and rare genotypes of gynogenetic triploid females, which is in line with the prediction of the Red Queen hypothesis.

The present study revealed that at the individual level high MHC diversity is not an important factor determining parasitism, using whichever of the two measures of individual MHC diversity – the number of expressed *DAB* alleles or the type of *DAB* lineage. High diversity in MHC IIB genes allows the recognition of more antigens derived from parasites in a population. However, many MHC variants in the individual will also result in self-reactive T-cell elimination. In line with this theory, Nowak *et al*. [43] predicted that an intermediate number of MHC alleles should be associated with minimal parasite load, which was supported by the study of three-spined sticklebacks (*Gasterosteus aculeatus*) by Wegner *et al*. [44]. Our study on gibel carp showed that higher parasite species richness is linked to the expression of the alleles of the *DAB3* lineage. As the common triploid genotype “A” represents the genotype of the *DAB3* lineage, this finding simply reflects the high parasite species richness of the most common genotype rather than the importance of the *DAB3* lineage for parasitism. Moreover, it supports the study of Lampert *et al*. [10] suggesting that a higher allelic copy number in clonal fish might not convey an immune advantage (measured by parasite load in our study).

Thus, the present study may indicate that parasitism is one of the plausible candidates for explaining temporal changes in the population character of gibel carp, from the period of its introduction into Czech rivers until today. The first invasive populations were triploid females with gynogenetic reproduction; their MHC genotypes were probably infrequent in the new invaded habitats. It is possible that triploid clones were able to become common and a potential target for parasite selection due to their high reproductive ability linked to gynogenesis.

What might explain the recent coexistence of gynogenetic and sexual forms of gibel carp? Hakoyama and Iwasa [45] showed that parasitism plays an important role in bringing about the coexistence of asexual and sexual forms in Japanese crucian carp (*Carassius auratus*) by giving a frequency dependent benefit to the sexual population. This supports the prediction that the coexistence of sexual and unisexual forms should be stabilized by parasites [5,13]. The high susceptibility to diseases in the unisexual form could compensate the two-fold cost of sex in the sexual form. As recent knowledge on the distribution of gibel carp in Central Europe shows that both sexual and gynogenetic forms coexist in many natural populations, we suggest that the higher parasite load in common gynogenetic triploid genotypes could represent one of the possible factors promoting the coexistence of this sexual-gynogenetic complex. Hakoyama *et al*. [3] showed that the high prevalence of *Metagonnimus* (Trematoda) in Japanese crucian carp (*Carassius auratus*) is in part due to the lower immune activity of the phagocytes in the gynogenetic form. Therefore, for further studies of gibel carp, we suggest analyzing measurements of specific (IgM level) and non-specific immunity (phagocyte activity) to investigate whether enhanced parasite load in gynogenetic females reflects an immune disadvantage. However, the coexistence of sexual and unisexual forms may also be maintained by male mate choice (e.g. [11,46]) or spatial and temporal extinction and recolonization (e.g. [47,48]), factors that have not yet been analyzed in *C. gibelio*. Moreover, for a better understanding of whether the coexistence of sexual and gynogenetic forms in the populations of gibel carp is stable, or whether the evolution of gibel carp populations is directed toward strictly sexual reproduction, long-term studies are needed. The latter possibility was demonstrated for sexual and asexual snails by Jokela *et al*. [48], where co-evolutionary dynamics predicted by the Red Queen hypothesis favor sexual reproduction in natural populations, i.e. the most common clones were almost completely replaced by initially rare clones, while the sexual form persisted throughout a long period of investigation.

## Conclusions

Our study investigated the genetic diversity of MHC class IIB genes and the selection mechanisms potentially driving MHC diversity in diploid-polyploid fish species with dual reproduction strategies. In mixed populations of gibel carp, where gynogenetic triploids coexist with sexually reproducing diploids, the genotype and allelic diversity is reduced in triploids compared to diploids, which is in accordance with the hypothesis of sexually-mediated selection increasing the MHC diversity and fulfils a prerequisite of the Red Queen hypothesis. We showed that the most common genotypes of gynogenetic triploids are the target of parasite selection. This finding also supports the prediction of the Red Queen hypothesis. On the other hand, the individual allelic copy number does not seem to be important for fish immunocompetence (measured by metazoan parasite infection). We propose that the evolutionary twofold cost of sexual reproduction in diploids is compensated by the limited ability of asexual forms to escape parasitism and, thus, that this process could facilitate the recent coexistence of sexual and gynogenetic forms of gibel carp.

## Methods

### Fish samples

A total of 92 individuals of gibel carp were caught by electro-fishing from the Soutok locality (Dyje River, Czech Republic) in July and August 2005 and 2006. A sample of blood for ploidy determination was collected from each individual by puncture of the caudal vessel using a heparinized syringe. Analyses of ploidy level were performed by means of computer-assisted microscopic image cytometry according to Flajšhans [49]. All specimens were dissected within 24 h. The spleen of each gibel carp was removed, transferred to 1.5 ml tubes with an aliquot value of RNA*later*^*TM*^ Storage Solution (Sigma-Aldrich) and stored at −80°C.

### MHC analyses

RNA extraction, reverse-transcription, and PCR were performed as described in Seifertová and Šimková [36]. The complete exon 2 of *DAB* genes (functional genes of MHC class II*B* in fish) was amplified using the forward primer FishIIBEx2–1 F (5’-CTGATGCTGTCTGCTTTCACTGGA-3’) and reverse primer FishIIBEx2–3R (5’-CTGCACATCAACACAGCTGGATG-3’) described in Ottová *et al*. [37]. The primers amplify the complete exon 2 of *DAB* genes representing the beta 1 domain (276 bp), and the short part of the exon 3 representing the beta 2 domain (48 bp). SSCP (single-stranded conformation polymorphism) analysis was used for the detection of different *DAB* sequence profiles in individuals based on the position, shape and number of chromatogram peaks, as detailed in Seifertová and Šimková [36]. On the basis of the SSCP results, the individuals with different SSCP patterns were then selected. The PCR products of each individual with a different SSCP pattern were amplified using non-labelled primers; the purification, cloning and sequencing that followed were performed as described in Seifertová and Šimková [36].

The new *DAB* alleles were deposited in EMBL under Accession numbers from HF934073 to HF934102. To eliminate PCR bias and random artefacts [50,51], the same criteria as detailed in Seifertová and Šimková [36] were applied. Nucleotide sequences of exon 2 were edited using Sequencher (Gene Code Corporation, Ann Arbor, MI) and aligned in BioEdit 7.0.9.0 [52] using Clustal W multiple alignment [53]. 26 out of 30 determined MHC sequence variants were found in at least two different individuals. The four remaining MHC sequence variants were identified in at least three clones obtained from each of two independent PCRs from the same individual.

### Genetic diversity of *DAB* sequences

The overall average nucleotide distances (calculated using Jukes-Cantor model) and amino acid distances (calculated using Poisson correction) in the *DAB* data set were computed in MEGA 5 [54] for the entire set of *DAB* sequence variants for diploids and triploids. Nucleotide and amino-acid distances (considered as a measure of MHC diversity) were also calculated for each individual fish expressing at least two *DAB* alleles. The relative rate of nonsynonymous (*d*_N_) and synonymous substitutions (*d*_S_) was calculated according to Nei and Gojobori [55], applying the correction of Jukes and Cantor [56] for multiple hits using MEGA 5 [54].

### Positive selection in *DAB* genes

The hierarchical likelihood ratio test implemented in ModelTest 3.6 [57] was used to determine the appropriate substitution model of sequence evolution that best fits the dataset. The ML trees were reconstructed in PAUP* 4.0b10 for Microsoft Windows 95/NT [58] using a heuristic search with a TBR (tree bisection reconnection) branch-swapping algorithm and used in the selection analyses performed in PAML 4.3 [59]. Analyses to detect selection on a site-by-site basis using a maximum likelihood approach in the program CODEML implemented in PAML were performed. Different models with and without selection incorporated were used to test for the presence of sites under selection. The models used the nonsynonymous/synonymous rate ratio (ω = *d*_N_ /*d*_S_) as an indicator of selective pressure on the protein. Put simply, values of ω < 1, = 1, and > 1 mean negative purifying selection, neutral evolution, and positive selection, respectively. As recommended by Yang [59], the following models were compared: M0 (one ratio) versus M3 (discrete model involving three sites classes for ω), M1a (nearly neutral) versus M2a (positive selection), and M7 (β model which uses beta distribution) versus M8 (β and ω ratio estimated from the data). A likelihood ratio test (LRT) statistic was used to assess the significance of the differences between models. The Bayes Empirical Bayes (BEB) method (implemented under models M2a and M8 only) was used to calculate the posterior probabilities (pP) for site classes and to identify sites under selection (the posterior means of ω for positively selected sites are > 1) following the recommendation of Yang [59].

### Parasite examination

Complete dissection of the fish was performed following the method of Ergens and Lom [60]. Fish were examined for all metazoan ectoparasites (Monogenea, Crustacea, Mollusca and Hirudinea) and endoparasites (Digenea, Cestoda, Acantocephala and Nematoda). The parasites were removed, fixed according to standard methods used in fish parasitology, and determined to species level using an Olympus BX50 light microscope. For each fish individual, parasite species richness and total parasite abundance (i.e. total number of parasite individuals in a given fish individual) were calculated. In addition, as *Dactylogyrus* species represent the most abundant parasite group, *Dactylogyrus* abundance (i.e. total number of *Dactylogyrus* individuals) was evaluated in this study. Parasite diversity was calculated using the Brillouin diversity index following Magurran [61].

### Statistical analyses

The effect of year of collection on the individual number of *DAB* alleles was tested using the non-parametric Mann–Whitney test. The data on nucleotide and amino-acid diversity were checked for normality. MHC diversity between two forms of gibel carp was compared using the t-test and the F-test of variance. Parasite abundance was log-transformed to fit the normal distribution prior to performing parametric statistics. The t-test was applied to analyze the differences in parasitism (1) between sexual diploids and gynogenetic triploids and (2) between triploids with the common genotype, triploids with rare genotypes, and diploids. Bonferroni correction was applied when using multiple t-tests.

Nested ANOVA was applied to analyze the effects of fish MHC and fish reproduction strategy (with MHC as a nested factor in reproductive mode) on parasite load (dependent variable). First, two nested ANOVAs were performed including the number of MHC alleles as a measure of fish MHC and parasite species richness or parasite abundance as a measure of parasite load. Next, two nested ANOVAs were performed including the type of MHC lineages as a measure of fish MHC and parasite species richness or parasite abundance as a measure of parasite load. Concerning the type of MHC lineage, three groups were distinguished as follows: (1) individuals expressing only the alleles of the *DAB1*-like lineage, (2) individuals expressing only the alleles of the *DAB3*-like lineage, and (3) individuals expressing the alleles of both lineages. Low MHC diversity is represented by the expression of one lineage, i.e. *DAB1* or *DAB3*; high MHC diversity is represented by the expression of two lineages, i.e. *DAB1* and *DAB3*. The Bonferroni post-hoc test was applied to test differences in parasite load between MHC groups. All statistical analyses were performed in Statistica 10.0 for Windows, StatSoft Inc.

## Competing interests

The authors declare that they have no competing interests.

## Authors’ contributions

AS designed this study and drafted the manuscript. MK and LV acquired fish and parasite data. MK and MV carried out the molecular genetic studies. AS, MK and MV analyzed the data. MK and LV have been involved in drafting the manuscript or revising it critically for important intellectual content. All authors read and approved the final manuscript.
